# Hypodermic Injection of Euclayptol in Phthisis

**Published:** 1887-02

**Authors:** 


Hypodermic Injection of Eucalyptol in Phthisis.—Roussel (*Allg. wiener Med.-Ztg.*) reports favorable results following this treatment; purulent sputa became mucous, the appetite and strength returned, and the cough was relieved.—*N. Y. Medical Journal.*

